# Developing an Ethically Acceptable Virtual Fencing System for Sheep

**DOI:** 10.3390/ani8030033

**Published:** 2018-02-27

**Authors:** Danila Marini, M. Dennis Meuleman, Sue Belson, T. Bas Rodenburg, Rick Llewellyn, Caroline Lee

**Affiliations:** 1 School of Environmental and Rural Science, University of New England, Armidale, NSW 2350, Australia; caroline.lee@csiro.au; 2 Commonwealth Scientific and Industrial Research Organisation, Agriculture and Food, Locked Bag 1, Armidale NSW 2350, Australia; dmeuleman@hotmail.com (M.D.M.); Sue.Belson@csiro.au (S.B.); 3 Behavioural Ecology Group, Department of Animal Sciences, Wageningen University, 6700 AH Wageningen, The Netherlands; 4 Adaptation Physiology Group, Department of Animal Sciences, Wageningen University, 6700 AH Wageningen, The Netherlands; bas.rodenburg@wur.nl; 5 Commonwealth Scientific and Industrial Research Organisation, Agriculture and Food, Locked Bag 2, Glen Osmond, SA 5064, Australia; Rick.Llewellyn@csiro.au

**Keywords:** virtual fencing, technology, welfare, associative learning, sheep management

## Abstract

**Simple Summary:**

Virtual fencing has the potential to be implemented in animal management systems where conventional fencing cannot be applied. Previous work has been conducted on cattle with virtual fencing technology that uses a collar that emits a warning audio when an animal approaches a GPS set boundary. If the animal continues walking towards the boundary an electric stimulus is applied. Less research has been conducted on sheep virtual fencing, so alternatives need to be explored to test the proof of concept on the application of virtual fencing with sheep. Using manually controlled training collars, 30 crossbred sheep were trained to respond to an audio cue in order to avoid receiving a low-level electrical stimulus.

**Abstract:**

To ensure animal welfare isn’t compromised when using virtual fencing, animals must be able to associate a benign conditioned stimulus with an aversive stimulus. This study used an associative learning test to train 30, four-year-old, Merino x Suffolk ewes, to associate an audio cue with an electric stimulus. Collars manually controlled by a GPS hand-held unit were used to deliver the audio and electric stimuli cues. For the associative learning, when sheep approached an attractant at a distance of three m from the trough, an audio cue was applied for one s. If the sheep stopped or changed direction, the audio cue ceased immediately and no electrical stimulus was applied. If the sheep did not respond to the audio cue it was followed by a low-level electrical stimulus. Approaches to the attractant significantly decreased from day one to day two. It took a mean of three pairings of the audio cue and electrical stimulus for a change in behaviour to occur, after which sheep that approached the attractant had a 52% probability of avoiding the electrical stimulus and responding to the audio cue alone. Further research is required to determine whether sheep can be trained to associate an audio cue with a negative stimulus for use in group grazing situations.

## 1. Introduction

There is increasing interest in virtual fencing systems to contain and control the movement of livestock. Unlike traditional fencing that uses a physical barrier which animals can see and interact with, virtual fencing relies on animals interacting with the fence through warning cues and a negative stimulus, such as an electrical stimulus. Virtual fencing systems offer potential benefits in animal management [1], including time and labour savings through the reduction in the erection and maintenance of standard and electric fences [2]. There are also potential environmental benefits through the protection of sensitive areas, wildlife conservation, and reduction in overgrazing [2,3].

To minimise welfare impacts on sheep when using a virtual fencing system, it is important that animals can control and predict when they are at risk of receiving the aversive stimulus i.e., sheep have the ability to understand when they are approaching the fence and can respond by staying within the fence line and avoid receiving the electrical stimulus. Previous research in cattle have shown associative learning to be an effective method of training cattle to a virtual fence [4]. To allow animals to make an association, two stimuli are presented in sequence. In the case of virtual fencing, it involves the use of a conditioned stimulus (for instance an audio cue) that is paired with an unconditioned stimulus (electrical stimulus). Further to this, the application of the audio cue and electrical stimulus should also incorporate the animal’s behavioural state to allow for the animal to recognise that its behaviour influences the imminent occurrence of a negative stimulus. Lee et al. [5] demonstrated that cattle were able to rapidly associate an audio cue with an electrical stimulus and were able to modify their behaviour through associative learning to avoid receiving the electrical stimulus. This has been proposed to be an ethical method of virtual fencing [4,5] as once the association is established, animals have the ability to avoid the fence by responding to the audio cue alone, much like they would learn to avoid touching an electric fence. However, it is important that the training method of associating the audio cue, electrical stimulus and animal’s behaviour are applied consistently, otherwise animals are at risk of becoming confused, affecting their ability to learn, and potentially negatively impacting their welfare [4].

The ability for sheep to learn a virtual fencing system is less clear. A study by Jouven et al. [6] found sheep could be readily contained by a virtual fencing system, however the effectiveness of the virtual fence decreased with the introduction of an attractant or an increase in the number of untrained sheep in the flock. It was also unclear whether the sheep had learnt to respond to the audio cue alone in this study, as learning was defined as successful containment for 30 min [6]. Brunberg et al. [7,8] also demonstrated that sheep could be trained to a virtual fence, with sheep displaying desired responses to the audio cue during training. The sheep in these studies were only given three opportunities to make an association and again the effectiveness of the virtual fence in containing the sheep was variable with further testing. It is clear that further research is needed to understand sheep responses to the audio and electrical stimulus cues.

There are challenges to developing commercial virtual fencing systems for sheep. The virtual fencing system for cattle is applied through the means of a collar worn by individual animals. Due to the large number of sheep within flocks, the cost of each animal wearing a device may be restrictive in commercial situations. In addition, the insulating properties of wool can prevent the electrodes of the virtual fencing device contacting the skin which prevents the electrical stimulus being applied. When combined, these challenges may be why less research has been conducted on virtual fencing in sheep to date [6,7,8] and the potential for ear-based devices continues to attract interest as a future platform for sheep [1].

To develop an ethical virtual fencing system for sheep, we must demonstrate that sheep can associate an audio cue with the electrical stimulus and learn to respond appropriately to the audio cue alone. This study uses manually controlled electronic collars to apply audio and electrical cues to assess the ability of sheep to learn this association during virtual fencing. The objective of this study was to develop a training method based on associative learning that enables the animal to recognise a benign cue as a sign of its imminent exposure to the aversive stimulus.

## 2. Materials and Methods

### 2.1. Ethical Statement

The protocol and conduct of the experiment was approved by The Commonwealth Scientific and Industrial Research Organisation (CSIRO), Chiswick Animal Ethics Committee under the NSW Animal Research Act, 1985 (approval ARA 16/15).

### 2.2. Equipment

Manually controlled dog training collars that are capable of administering an audio and an electrical stimulus (Garmin TT15, Garmin Ltd., Kansas, KS, USA) were used in this study. The electrical stimulus was applied through two probes on the collar, which sat directly on the sheep’s skin. The collars required no modification for the probes to have contact with the sheep. To provide optimal contact between the collars probes and the sheep’s skin, the wool around the sheep’s neck was removed the collars were fitted so that the probes were contacting the underside of the neck. The collars were used in combination with a Garmin GPS hand-held unit (Garmin Alpha 100, Garmin Ltd., Kansas, KS, USA), which when prompted delivered an audible (70–80 dB, 2.7 kHz) and an electrical stimulus, which was set to level 4 (320 V, 20 us, with 16 pulses delivered per s, with no resistance), to the collar. The audible emitted from the collars were within normal sheep hearing range [9]. The electrical output reported does not take into account the electrical impedance of the sheep’s wool and skin and is a lower level than has been used on sheep previously [7,10]. If the collar did not function as expected during tests, such as when no response was shown, then the sheep was returned to the pen and the collar was adjusted to ensure correct fit and the test re-run. In the periods between the tests, the sheep were fitted with a dummy collar matching the shape and weight of the Garmin collars. For all of the experiments, a video camera (Sony Handycam HDR-XR260E, Sony Electronics Inc., San Diego, CA, USA) was used to record behaviour during the test.

### 2.3. Pilot Trial

To determine an appropriate electrical stimulus to be used in this experiment, pilot tests were run with 30 different sheep. During the tests the intensity of the electrical stimulus was modified and the animals behavioural response to the stimulus was observed (data not presented). The stimulus that was required needed to be adverse enough to the animal that it would prevent them from reaching a trough, but not so adverse that it would cause negative behavioural responses (e.g., falling over, running around the paddock). The stimulus level that was chosen was sufficient to stop all of the animals tested from reaching a feed trough.

### 2.4. Animals

Thirty, four year-old Merino cross White Suffolk ewes (66 ± 0.8 kg body weight) were used in the experiment. For identification, all of the sheep were randomly assigned a number which were marked onto their flanks using wool paint (Si-Ro-Mark, Cox Agri, County Durham, UK). The experiment was conducted at Commonwealth Scientific and Industrial Research Organisation in Armidale NSW, Australia. Throughout the experiment the sheep were provided with water ad-libitum and kept on pasture. They were also supplemented with sheep pellets on a daily basis, this was done to habituate the sheep to the pellets as well as the presence of humans.

### 2.5. Experimental Protocol: Associative Learning

The sheep were first trained to approach a feed trough and eat a food reward (total mixed ration pellets, Ridley Agriproducts, NSW, Australia). All of the sheep were trained twice a day for five days, in a small grassed paddock (25 × 25 m). A trough containing pellets was placed in the centre of the paddock, approximately 12 m from the entrance point, the handlers stood near the test entry and were visible throughout the test. On day one, sheep were released into the paddock in groups of three, this was reduced to two sheep for day two, and on day three onwards sheep were trained individually. Once sheep had reached the trough they were allowed to feed for one min, they were then quietly moved to the test exit. During the training and testing sessions an elevated viewing platform was positioned approximately ten m outside of the paddock, to habituate the sheep to its presence. All animals reached the trough during training.

**Figure 1:**
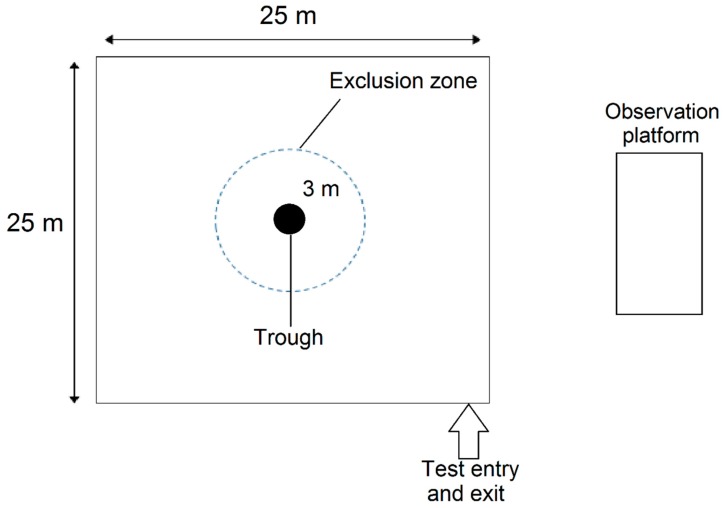
Schematic of the test paddock used for experiment 1 and 2, the area was approximately 25 × 25 m, with the trough placed in the centre. An exclusion zone three m around the trough was discreetly marked with rocks.

Testing occurred the week after training using the same design as for training, except that a three m exclusion zone was marked around the trough with small rocks as a visual aid for the operators applying electrical stimuli to sheep entering the exclusion zone (details below), (Figure 1). Prior to the test, each animal was fitted with the Garmin collar. Next, an individual sheep was released into the paddock and the test commenced. As the sheep approached the exclusion zone, a one s audio cue was delivered. If the sheep displayed either of the following responses: (1) stopping, (2) turning away or backing up, (3) running forward, or (4) moving out of the exclusion zone, the audio cue was ceased before one s elapsed. If the sheep failed to respond to the audio cue after one s, then an electrical stimulus was immediately applied for a maximum of one s. If the sheep turned towards the trough and approached the exclusion zone again then another audio was given followed by the electrical stimulus. If the animal was still within the exclusion zone then the cues were given as soon as the animal turned towards and approached the trough. The application of an audio cue upon interacting with the fence is defined as one event, the events may or may not be paired with an electrical stimulus.

The animal’s behavioural response to both the audio cue and the electrical stimulus was recorded throughout the test by an observer as well as a video camera to verify behaviours. The behaviours that were recorded included movement behaviours, such as turning, continuing, and stopping. Other behaviours, such as headshaking, jumping, and rearing, could occur in addition to the animals’ movement and were also recorded. The test ended after five min or after a maximum of five electrical stimuli were applied. Testing was conducted once a day for five days.

The sheep dramatically reduced their number of approaches to the trough after day two. To encourage sheep to continue to approach the trough, on the third day, following associative testing, the sheep were allowed to approach the trough containing feed without receiving any cues or stimuli. On the fifth day, the feed trough was relocated within the paddock, and sheep were again tested in the associative learning test.

### 2.6. Statistical Analysis

The statistical analysis software R (The R Development Core Team, Version 3.3.3, R Foundation for Statistical Computing, Vienna, Austria.), package *pgirmess* [11] was used for analysis. The data for the number of approaches was not normally distributed and was analysed using a Kruskal-Wallis test. Continuing and turning behaviours in response to the audio cue were compared using a proportions test. The sheep’s movement behaviours in response to the stimulus before and after learning was analysed using a fishers exact test, other behaviours in response to the stimulus were analysed using a chi-square test. *p* < 0.05 was considered to be statistically significant and 0.1 > *p* > 0.05 was considered a statistical tendency.

For the associative learning test, a logistical regression was used to evaluate the learning period in which sheep were able to respond to an audio cue to avoid an electrical stimulus. A logistic curve was fitted to the data using the non-linear least squares function, the method in which the data was analysed has been previously described in Lee, et al. [5]. The application of the audio cue and electrical stimulus from each animal (i) were paired in sequence to create a binary variable, where the audio event number X_ij_ and a binary variable Z_ij_ which is zero if the sheep received during the interaction event an audio not followed by an electrical stimulus and one if the audio was followed by an electrical stimulus. A general logistic curve of the form of

$$\pi =a+\frac{c}{1+\exp\left(-b\left(x-m\right)\right)}$$

was fitted for 30 sheep, where π is the probability that Z_i_ = one, a is the lower asymptote, a + c is the upper asymptote, b is a slope parameter, m is the point of inflection.

In relation to learning behaviour, the slope of the logistic regression is related to rate of behavioural change. A negative slope indicates that a proportion of the animals receiving an electrical stimulus following an interaction with the fence decreases with repeated interactions. The upper asymptote in the proportion of naïve animals receiving an electrical stimulus, the lower asymptote is the proportion of animals that still receive an electrical stimulus after behavioural change. No constraints were applied in fitting the curve, allowing for the slope parameter to be greater than zero and the asymptote to be outside the meaningful range of zero to one. The point of inflection is the number of interaction it takes for half of the animals change their behaviour, which is the change in proportion between the upper and lower asymptote. In addition to the logistic regression the proportion of audio and stimulus, before and after learning occurred, was analysed using a chi-square test.

## 3. Results

There was a significant difference in the number of sheep approaching and entering the exclusion zone over the five day testing period (*H* (4) = 80, *p* < 0.05). A majority of approaches occurred on day 1, when all 30 sheep approached and received an audio cue, with a mean of three. This significantly decreased on day 2 (*difference* = 71.3), with a critical difference of 28.3 (α = 0.05), with only eight sheep approaching and receiving an audio cue. The number of approaches on days 3 to 5 did not differ from day 2 (*difference* < 10, critical difference = 28.6, α = 0.05).

**Figure 2:**
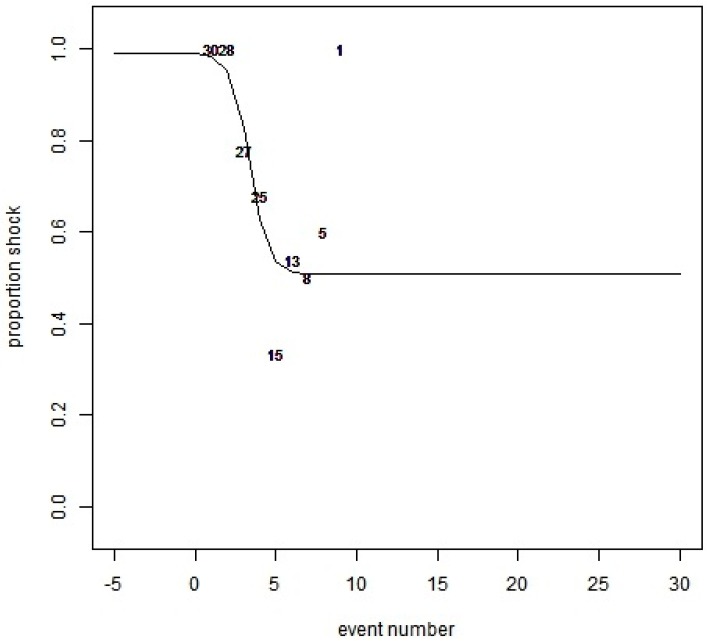
Logistic curve for the associative learning trial. Y-Axis is the proportion of animals that receive a stimulus following an audio cue. X-Axis is number of attempts to enter the exclusion zone throughout the entire testing period. The numerals are the number of animals that approached the exclusion zone for that event number. Event is defined as one interaction with the virtual fence determined by application of an audio cue.

The logistic model (Figure 2) fitted the data well, as seen by the observed proportions being close to the fitted line. The upper asymptote is almost equal a 1.0, representing that all animals that entered the exclusion zone received an electrical stimulus following the audio cue on their first interaction. The number of animals that entered the exclusion zone significantly dropped to only half of the tested animals by attempt five. Of the 15 animals that approached and entered the exclusion zone for a fifth time, only 33% received an electrical stimulus (five animals). The lower asymptote is 0.48, therefore, even after several interactions with an audio paired with a cue, animals entering the exclusion zone would still have a 48% probability of receiving an electrical stimulus. The estimated parameters for the logistic regression is presented in Table 1.

There was a significant association of learning and whether an animal would receive a low-level electrical stimulus χ^2^ (1) = 3.7, *p* = 0.05. Based on the odds ratio, the odds of a sheep receiving a stimulus were 0.611 (0.35, 1.04) times higher before the learning period, the proportion receiving a stimulus was 47% before learning, and 35% after. Out of the 30 animals that were tested 63% responded to the audio cue alone during testing. Individual interaction with the fence varied, with one sheep only having one interaction and another having nine interactions.

The date in Table 2 and Table 3 the data is split into events (interaction with the fence) 1 to 3 and 4 to 9, as it is indicated by the logistic regression the change in behaviour that occurred after three events. There is a significant difference in the proportion of animals that stopped or turned in response to the audio before and after learning (χ^2^ (1) = 20, *p* < 0.05, Table 2). The proportion of animals that stopped or turned away from the virtual fence on the audio cue prior to learning was 4.7% after learning this increased to 31%.

There was variation between sheep and the behaviours that they displayed in response to the electrical stimulus. There was a significant difference between the movement behaviours observed before and after learning (Fishers exact test, *p* < 0.05). Following learning, animals continuing forward into the exclusion zone reduced to 2.7% and moving back was the most common behaviour observed 86.5%. The other behaviours displayed by sheep, jumping, rearing, and headshaking, did not vary before and after the learning phase (χ^2^ (2) = 3.18, *p* = 0.2, Table 3).

## 4. Discussion

In our study, we determined that a low-level electrical stimulus was effective at preventing the sheep from reaching an attractant. Importantly, we demonstrated that sheep were able to associate an audio cue with an imminent aversive cue and respond to the audio cue alone. These two criteria, a minimal effective aversive stimulus and learning to respond to a benign cue should enable the development of an ethically acceptable virtual fencing system for sheep.

The methods that were used for associative training enabled sheep to associate the audio cue with an electrical stimulus, with half of the learning occurring after receiving three pairings of an audio with the electrical stimulus. After five interactions, 10 of the 15 animals that approached the trough withdrew from the exclusion zone in response to the audio cue alone. The previous studies conducted with sheep [6,7,8] used different training protocols, making it difficult to compare the ability of sheep to learn to turn away on an audio cue. Brunberg et al. [8] found that 37.5% learnt to respond to the audio, however, they only allowed animals to interact with the virtual fence around a feed attractant three times. In our study, at the third interaction with the virtual fence 22% of sheep successfully responded to the audio cue alone. The logistical regression indicates that learning occurs after the third interaction. In our study, when sheep were allowed to interact with the fence beyond three attempts, the proportion of sheep that successfully responded to the audio cue increased to 67% at the fifth interaction. Similar results have also been observed in cattle, with only 31% of cattle responding to audio alone on the third interaction, then increasing to 56% by the fifth interaction [5].

The logistic regression indicates that even after learning occurs, 48% of the animals that approach the virtual fence would still receive an electrical stimulus. There was also one animal that still received an electrical stimulus, even on its ninth approach. This highlights the large variability between individual animals learning ability, aligning with results of previous studies [1,4,5,6,7,8]. The variation observed in learning could have been due to the design of the test as sheep were tested in isolation, which can be more stressful. Stress has been shown to affect learning ability [12] and cause learning deficits in sheep [13]. Importantly, differences may exist between animals in their sensitivity to electrical stimuli and in their capacity to learn the association between an audio cue and an imminent electrical stimulus.

The associative training procedure was designed to encourage sheep to repeatedly approach the trough in order for enough interactions to occur for an association between the audio cue and the electrical stimulus to form. However, by the fifth interaction, only half of the animals that were tested approached the trough. Allowing for sheep to obtain a feed reward from the trough halfway through the training period and changing the location of the trough was not enough to restimulate approaches. Sheep have good spatial memory [14] and are able to learn the location of feed within a landscape, with memory being enhanced through the additional presence of visual cues [15,16]. The use of a feed trough as the attractant could have provided the sheep with a visual cue to associate with the electrical stimulus. This could have assisted the sheep with learning to pair the audio cue and the electrical stimulus [5], but could have also led to a reduction in approaches to the feed trough over the testing period.

As virtual fencing uses negative reinforcement through the use of an aversive stimulus it is important to find an appropriate stimulus level to deter animals without compromising welfare. Previous studies have shown that the application of electrical stimuli can have a negative effect on welfare [17]. This is especially the case when the animal is unable to predict, and therefore unable to have the ability to control the occurrence of the negative stimulus [18]. When using low energy electrical stimuli on cattle, Lee et al. [19] found that the cattles’ stress response was similar to being restrained in a crush. Our study only observed the behavioural responses of sheep to the application of the electrical stimulus and ensured that animals did not respond in an excessively negative manner. Further research needs to be conducted to evaluate the effect of a low electrical stimulus on sheep welfare.

## 5. Conclusions

Using associative learning to train sheep to a virtual fence, the sheep were able to learn to respond to a benign audio cue in order to avoid the aversive electrical stimulus after only three prior experiences with the fence. Importantly, there was high variability between individual animals in their interactions with the fence and their associative learning ability. The ability to virtually fence sheep would allow farmers to have the potential to manage grazing efficiency particularly in large fields where intensive targeted grazing can be used for weed and cropping management. Further research is needed to overcome the challenges in developing a feasible virtual fencing system for sheep. In addition, research is also required to determine the impact that an electrical stimulus has on sheep welfare and how sheep in a flock learn to associate audio cues with a negative stimulus for use in virtual fences deployed across a diversity of environmental contexts. 

## Figures and Tables

**Table 1:** Estimated parameters for the logistic regression curves for 30 sheep during associative learning. The upper asymptote indicates the proportion of naïve animals that received a stimulus during these events. The lower asymptote is the proportion of animals that continued to receive a stimulus. The difference between these is tested for significance. The point of inflection is the mean number of attempts it takes for half of the learning to occur. The slope indicates the speed of transition from the upper to lower asymptote.

Upper Asymptote	Lower Asymptote	Sig Diff	Point of Inflection	Slope	Fit
0.99	0.48	< 0.05	3.36	−1.75	0.15

**Table 2:** Count of behaviours displayed by sheep in response to the audio cue (*n* = 152) during associative training, *n* is the number of approaches to the exclusion zone. Proportions are displayed in brackets.

Behaviours	Audio Cue		
	Event 1 to 3 (*n* = 85)	Event 4 to 9 (*n* = 67)	Total Count
Continue	81 (95.3%)	46 (68.7%)	127
Turn or stop	4 (4.7%)	21 (31.3%)	25

**Table 3:** Count of the behaviours displayed by sheep following a low-level electrical stimulus (*n* = 114) during associative training, *n* is the number of entries into the exclusion zone. Proportions are displayed in brackets. Animals were able to display more than one reaction in one interaction with the virtual fence, such as a head shake and run forward.

Behaviours	Electrical Stimulus		
Movement Behaviours	Event 1 to 3 (*n* = 77)	Event 4 to 9 (*n* = 37)	Total Count
Back	34 (44.1%)	32 (86.5%)	66
Continue	15 (19.5%)	1 (2.7%)	16
Turn	28 (36.4%)	4 (10.8%)	32
Other behaviours			
Jump	42 (56.0%)	14 (38.9%)	56
Rear	17 (22.7%)	13 (36.1%)	30
Headshake	16 (21.3%)	9 (25.0%)	25
